# Supplementary material for: Are carbon emissions trading and green financial instruments synergistic? -Comprehensive quantitative research based on content analysis
Source: PLoS One. 2024 Mar 7;19(3):e0298601. doi: 10.1371/journal.pone.0298601 (PMC10919601; doi:10.1371/journal.pone.0298601)
Supplement: S1 Data — (ZIP) [file pone.0298601.s001.zip › Data/地级市Z_F工作报告.zip!/╜¡╦╒/╤∩╓▌╩╨/└┤╫╘╣1⁄2╓┌║┼í╛┬φ┐╦╩2╛▌═°í┐_2017.docx]

各位代表：

现在，我代表扬州市人民政府向大会报告工作，请予审议，并请市政协委员和其他列席人员提出意见。

2016年和本届政府工作回顾

过去的一年，面对严峻复杂的宏观形势和艰巨繁重的发展任务，我们在中共扬州市委的正确领导下，认真贯彻落实习近平总书记系列重要讲话特别是视察江苏重要讲话精神，较好地完成了七届人大五次会议确定的各项目标任务。全年实现地区生产总值4449.4亿元，增长9.4%；一般公共预算收入345.3亿元，增长2.5%；全社会固定资产投资3288.7亿元，增长15.3%；城乡居民人均可支配收入达35659元和18057元，分别增长8.2%、8.7%。期末城镇登记失业率1.88%。

1.产业结构持续优化

工业经济加快转型。规上工业增加值增长9.2%。五大主导产业产值6816亿元，占规上工业67.5%，其中机械、汽车产业产值分别达3521亿元、1326亿元。新增国家高新技术企业102家，高新技术产业、战略性新兴产业产值分别占规上工业44.7%、43.6%。新开工工业重大项目54项，新竣工投产46项。新建省级以上智能车间12个。建筑业产值3350亿元，增长8%。

服务业发展全面提速。服务业增加值占GDP比重45%。软件和互联网产业业务收入841亿元，增长40.2%；从业人员突破3万人。旅游业总收入860亿元，增加值占GDP比重7.5%。新增4A级物流企业1家、3A级3家。新增上市公司3家、“新三板”挂牌公司20家，新设金融机构7家，直接融资318.9亿元。科技服务业收入增长15.3%。文化产业增加值占GDP比重4.8%，新增文化服务业列统企业76家。全市电子商务交易额550亿元，增长50%以上。建成运营城市商业综合体5个。社会消费品零售总额增长9.9%。

现代农业稳步推进。虽然遭遇不利天气影响，粮食总产量仍稳定在30亿公斤左右。积极化解农民卖粮难，粮食收购总量21.2亿公斤。开工建设农业重大项目32个，新建高标准农田20.45万亩、设施农（渔）业13.9万亩。积极抵御流域性洪水，防洪抗灾取得胜利。更新改造小型泵站521座，新建防渗渠道400公里，水利事业持续发展。在全省率先开展粮食生产全程机械化创建工作，农业机械化水平85.1%。创成“三品一标”农产品174个。新增家庭农场698个、国家级农民专业合作示范社4个。在全省率先实现农产品产地检测监控乡镇全覆盖。江都创成省级农产品质量安全县。仪征建成全省首个国家农业社会化服务标准化示范区。省级以上农业龙头企业销售增长10.4%。农业开发、供销、气象等在“三农”工作中发挥重要作用。

2.发展活力不断增强

科技创新成效显著。成功入选全国小微企业创业创新基地城市示范。创成全国质量强市示范城市，获批筹建国家玉器产品质量监督检验中心。扬州国家高新区正式挂牌，生态科技新城和高邮湖西新区获批建设省级高新区。新建科技综合体和众创空间106万平方米，入驻企业突破1200家，新增国家级孵化器3家、省级以上众创空间11家。新引进知名高校研发平台、央企和世界500强创新中心46家。新建省级以上“三站三中心”74家。20个项目获批省重大科技成果转化专项资金1.68亿元。341名高层次人才入选省级以上重点人才引进培养计划。

改革攻坚持续深化。跨江融合发展综合改革试点取得新进展。供给侧结构性改革稳步推进，出台“1+5”实施意见，化解造船产能83万载重吨，消化煤炭产能28万吨，全市商品住房库存去化周期降至7.4个月，房地产市场平稳健康发展。全面实施“五证合一、一照一码”。推进国有资产监管全覆盖。“营改增”改革全面推开。推广政府与社会资本合作模式，实施PPP项目29个。军民融合产业深度发展，新增军民融合企业27家。

开放开发步伐加快。新落户世界500强及跨国公司项目6个。实际利用外资12亿美元，增长41.9%。完成外经营业额8.19亿美元、境外投资额6亿美元。扬州出口加工区获批国家综合保税区，维扬开发区创成国家生态工业示范园区。省级以上开发区承接上海、苏南园区亿元以上项目25个。扬州泰州机场升级为国际机场，新开辟国际航线5条。

3.城乡面貌明显改变

城市功能更加完善。宁启铁路开启动车。连淮扬镇铁路扬州站站场开工建设。邗江南路全线通车，城市南部快速通道、金湾路、S611等加快推进。西区新城、生态科技新城、广陵新城建设明显提速。完成国庆路南段、渡江路等骨干道路整治。改造区域供水支管网220公里。推进10大生态中心和城市公园体系建设，新改扩建公园626万平方米。成功申办省园博会和世界园博会。

新型城镇化加快推进。制定推进乡镇差别化发展指导意见。加快实施居住证制度，基本实现户口迁移“零门槛”。市县乡村四级就业服务平台实现全覆盖。基本公共服务水平不断提高，合理配置基础教育资源，来扬务工农民工子女就学享受市民待遇。建成农村区域性医疗卫生中心5家。11个村入选省级美丽乡村建设试点。全市城镇化率达64.4%。

生态文明建设成效彰显。整治挥发性有机物污染企业30家；淘汰10蒸吨以下小型燃煤锅炉1296台套；淘汰黄标车7813辆、老旧汽车6206辆；取缔露天烧烤点65个。市区空气优良率71.6%。完成6条市区河道整治。实行地表水“断面长制”，9个国家考核断面水质、11个城市集中式饮用水水源地水质全部达标。全市成片造林2.5万亩，恢复湿地5650亩，创成省级绿化示范村56个。古运河成功创成国家级水利风景区。率先启动江淮生态大走廊规划建设，并上升为省级战略。

4.人民生活日益改善

民生工程扎实推进。完成农产品专项抽检1500批次，新改建蔬菜基地2052亩。市区新改建农贸市场12个，取缔马路市场20处。新改建公共停车场3座、公厕76座。整治翠岗、康乐、宝带等老旧小区72万平方米，实施公房解危3.6万平方米，完成棚户区改造2411户。新辟优化公交线路26条，主城区公交分担率达26.6%，镇村公交覆盖率80%。新改建农村道路206公里、危桥65座、危房1260户，完成渔民上岸安居1432户。疏浚县乡骨干河道81条。实施经济薄弱村物业增收项目73个。

城乡居民收入稳步提高。全市新增城镇就业7.2万人、农村劳动力转移就业1.6万人。全年创业培训1.3万人，扶持成功创业7231人。培育生产经营型、专业技能型和专业服务型农民2.1万人。市区、县（市）最低工资月标准分别提高至1770元、1600元。全市35%的人均年收入低于7000元农户实现脱贫。

社会保障体系日益健全。全面实施城镇居民大病保险和城镇职工大病补充保险，为4300多名参保人员报销大病费用3400多万元。新型农村合作医疗人均财政补助提高至425元，政策范围内报销比例提高至76%。新增养老保险参保1.24万人、医疗保险3.19万人、工伤保险3.75万人、生育保险3.57万人。企业、机关事业退休人员基本养老金总体上调6.5%。城乡居民基础养老金月最低标准调至115元。实施医疗救助近11万人次2900万元。

5.社会发展和谐繁荣

社会事业蓬勃发展。全市改造中小学校舍10.2万平方米。新创省义务教育现代化学校55所。高考普通类本一本二上线人数增幅全省第一。市特殊教育学校新校区交付使用。扬州技师学院建成国家职教示范校。广陵学院新校区、江苏旅游职业学院开工建设，提标建设扬州老年大学。成功承办第六届江苏书展和首届江苏原创戏剧节双年展。高邮获批国家历史文化名城。新增市第一医院、市中医院等医疗联合体。人口计生指标完成情况全面好于省定标准。省运会筹备工作有序推进。新建更新健身设施1700余件。成功举办第六届全球残疾青少年IT挑战赛。台湾事务、外事、侨务、科普、档案和工会、青年、妇女、儿童、关心下一代、老龄、慈善、红十字等事业取得新进步。

社会治理有力有序。建成市综治法治指导协调中心，市县乡村社会管理服务平台四级贯通。提请市人大常委会审议地方性法规2项，出台《扬州市城市市容和环境卫生责任区管理办法》和《大运河扬州段世界文化遗产保护办法》2部政府规章。3个社区创成全国和谐社区建设示范单位。全市7个法律援助中心全部创成省级示范窗口。获评全国创新社会治理优秀城市，广陵区“志愿服务文明积分”项目获评全国创新社会治理优秀案例。蝉联全省法治城市先进市。荣膺全国“六五”普法先进市称号。文明城市常态化长效化管理成效显著。

社会大局保持稳定。市区技防城建设通过省验收，公众安全感达95.8%。开展危化品专项整治，建立安全生产监管执法平台，安全生产形势总体平稳。升级“阳光信访”信息系统，信访事项办结率97%、积案化解率89%。民族、宗教、仲裁、应急等工作取得积极成效。

6.政府自身建设不断加强

行政行为规范有序。自觉接受市人大及其常委会的法律监督、工作监督和市政协的民主监督，积极开展政府协商，广泛听取各民主党派、工商联、无党派人士和社会各界意见。市人大代表议案、建议338件，政协提案337件全部办复。在市级37个部门推行“双随机一公开”抽查机制。启动重大政策举措第三方评估工作。创成省行政复议工作规范化建设示范单位。

服务效能不断提升。加快职能转变，编制行政权力标准化清单和办事指南，实现行政权力“三级四同”。完善“一办两中心”服务体系，部门对窗口授权率80%以上。探索并联审批、多图联审、多评合一等举措，为企业提供全流程精准化服务。建成并开放全市政务数据服务网。“扬州发布”“扬帆”上线运行。

廉政建设得到加强。深入学习贯彻中央新近颁布的各项准则、条例。扎实开展“两学一做”学习教育。坚持市政府常务会学法学纪制度。落实党风廉政建设责任制，严格执行中央“八项规定”，严厉查处“四风”问题，努力营造风清气正的良好氛围。

各位代表，本届政府履职以来，认真学习贯彻党的十八大和十八届三中、四中、五中、六中全会精神，坚持“五大发展理念”，统筹做好稳增长、促改革、调结构、惠民生、优生态、防风险等各项工作，实现“十二五”顺利收官、“十三五”良好开局。可以说，过去五年是扬州综合实力提升最快、城乡面貌变化最大、人民群众得实惠最多的时期之一。

五年来，我们始终以质量效益为导向，发展水平实现新提升。连续3年制定实施服务企业“2号文件”，推动实体经济做大做强。2016年地区生产总值、一般公共预算收入分别是2011年的1.69倍、1.58倍，人均GDP在苏中苏北率先超省均。全市规上工业总产值迈入万亿元门槛；高新技术产业产值、服务业增加值均比2011年实现翻番。全社会固定资产投资年均增长17%以上，沿江100亿元、沿河50亿元重特大项目实现全覆盖。

五年来，我们始终以创新驱动为引领，改革开放迈出新步伐。加快推进科技创新，荣获国家创新型试点城市，创成国家高新区，获批国家现代农业示范区、国家农业科技园区，建成8个国家特色产业基地和12个省级科技产业园。深入开展跨江融合发展综合改革试点，所有省级以上开发园区均与上海、苏南开发区结对共建。医药卫生、食品药品监管、城市水务管理体制改革等顺利推进。供给侧结构性改革取得阶段性成效。推进更高水平对外开放，累计实际利用外资75.2亿美元，落户世界500强及跨国公司项目39个。

五年来，我们始终以文化生态为特色，名城建设形成新格局。建成文博场馆120家，7家文化馆全部跻身国家一级馆，科技馆获批省科普教育基地。原样复制《四库全书》。编制发行《扬州市志（1988-2005）》。牵头沿线城市成功申报中国大运河为世界文化遗产。基本建成9大生态中心，80个生态体育休闲公园建成开放。创成100个三星级康居乡村。荣获国家生态市、全国国土资源节约集约模范市。以纪念建城2500周年为契机，滚动推进30项重大城建工程。扬州泰州国际机场、瘦西湖隧道、新万福路和万福大桥等标志性工程建成并投入使用，连淮扬镇铁路扬州段、城市南部快速通道全线开工。全市配电自动化实现全覆盖。五年实施城建项目1400个，累计完成投资1830亿元。

五年来，我们始终以为民惠民为根本，民生工作再上新台阶。每年制定民生工作“1号文件”，新增财力70%用于民生。大力实施“八老”改造、“1161”菜篮子工程、“115”鲜奶工程，市区三区社会保险和民政福利“同城同步同标”，区域供水在苏中苏北率先全覆盖。持续推进“不淹不涝、清水活水”城市建设，实现主城区道路渍涝全消除和市区骨干河道活水全覆盖。2016年城镇、农村常住居民人均可支配收入分别是2011年1.59倍和1.66倍。全面完成人均年收入5000元以下低收入农户脱贫目标。

五年来，我们始终以共建共享为追求，社会建设取得新成效。深入推进平安法治建设，公众安全感连续13年超过95%，连续9年被评为全省社会治安安全市；荣获全国首批法治城市创建活动先进单位。建成市县乡村四级文化共享工程服务网络。各地均创成国家义务教育发展基本均衡县（市、区），全市高考成绩稳居全省第一方阵，成功引进南邮通达学院。创成省体育强市和首批公共体育服务体系示范区。城乡“15分钟健康服务圈”和“15公里半径医疗急救圈”基本形成。国防动员和后备力量建设不断加强，实现全国双拥模范城“七连冠”。

各位代表，以上成绩的取得，是全市人民在市委正确领导下团结拼搏的结果，是市人大、市政协及社会各界大力支持的结果，是历届政府接力耕耘、历任班子励精图治的结果。在此，我谨代表市政府，向全市人民，向市人大代表、政协委员，向各民主党派、工商联、无党派人士、各人民团体，向驻扬部队、驻扬单位，向所有关心、支持、参与扬州建设和发展的同志们、朋友们，表示崇高的敬意和衷心的感谢！

在肯定成绩的同时，我们也清醒地看到存在的问题，主要是：实体经济困难较多，转型发展、提质增效任重道远，创新驱动能力有待增强；沿江沿河地区发展不平衡的问题比较突出；居民收入持续较快增长难度加大，扶贫攻坚工作仍需继续努力；公共服务资源配置不够均衡，民生工作与群众的期望还有差距；社会治理和城市管理还存在薄弱环节；政府职能转变尚未完全到位，干部能力作风及政府自身建设需要进一步加强。对此，我们必须高度重视，采取更加有力措施加以解决。

今后五年政府工作指导思想、奋斗目标和主要任务

今后五年政府工作的指导思想是：紧密团结在以习近平同志为核心的党中央周围，高举中国特色社会主义伟大旗帜，以马克思列宁主义、毛泽东思想、邓小平理论、“三个代表”重要思想、科学发展观为指导，深入贯彻落实习近平总书记系列重要讲话特别是视察江苏重要讲话精神，紧紧围绕“五位一体”总体布局和“四个全面”战略布局，紧扣“两聚一高”，办好“十件大事”，以“迈上新台阶、建设新扬州”为主题，全面推进“经济强、百姓富、环境美、社会文明程度高”的发展进程，奋力谱写好中国梦第一个百年梦想的扬州篇章。

今后五年的奋斗目标是：

——经济实力再上台阶。坚持“稳中求进”，奋发有为，持之以恒做强实体经济。GDP年均增长8%左右，人均GDP稳定超省均。固定资产投资年均增长12%以上，其中工业投入占比55%以上。到2021年，服务业增加值占GDP比重50%以上。

——创新动能显著增强。持续“聚力创新”，突出企业主体，着力打造高水平创新平台。强化人才培育、核心技术攻关、科技成果转化和创新生态构建，打造先进制造业、现代服务业和战略性新兴产业为支撑的现代产业发展新格局，力争早日成为区域性创新创业中心。到2021年，主要创新指标达到或超省均；高新技术产业、战略性新兴产业产值分别占规上工业产值比重48%、45%。

——生态文明特色彰显。牢固树立绿色发展理念，走生产发展、生活富裕、生态良好的文明发展之路，打造美丽中国的扬州样板，把江淮生态大走廊建设成为清水大走廊、绿色大走廊和安全大走廊，注重生物多样性保护，争创国家生态文明建设示范市。五年新增植树2500万株，林木覆盖率提升至24%。

——城市品质全面提升。以筹备省运会、省园博会、世界园博会为契机，进一步优化城市空间布局，凸显“人文、生态、精致”城市特质，打响宜居、宜业、宜创、宜游城市品牌，不断提升城市知名度美誉度，加快建设古代文化与现代文明交相辉映的名城。到2021年，文化产业增加值占GDP比重6%以上。

——人民生活持续改善。突出“聚焦富民”，深入推进全民创业，到2021年力争城乡居民收入达到省均水平；进一步推动城乡居民“文起来、动起来、乐起来”，努力打造健康中国的扬州样本，倾力建设群众幸福指数高、获得感强的快乐家园。

实现“迈上新台阶、建设新扬州”目标，需要政府有更高的站位、更实的举措、更大的作为，一张蓝图绘到底、点点滴滴抓落实。具体做到“四个坚持”：

一是坚持把建成全面、高水平、人民群众认可的小康社会作为建设新扬州的首要任务。始终遵循以人民为中心的发展思想，贯彻落实习近平总书记提出的“七个更”工作要求。强化富民增收造血功能，积极鼓励创业、促进就业，五年新增城镇就业25万人、扶持自主成功创业2.5万人。城乡基本养老保险和医疗保险覆盖率均达98%以上。深入推进教育现代化建设，总体实现省定教育现代化指标体系要求。居民健康主要指标达到国际先进水平。完善立体化信息化社会治安防控体系，人民群众安全感持续增强。

二是坚持把践行创新、协调、绿色、开放、共享的发展理念作为建设新扬州的根本路径。持续推进创新发展，加快形成以创新为引领的经济体系和发展方式。聚焦发展软件和互联网、智能装备制造、汽车及零部件等优势产业，改造提升成型机械、车船装备等传统制造业，大力发展新能源、半导体照明、新一代信息技术等战略性新兴产业。全力创建国家创新型城市，五年新增发明专利授权量3000件、创省级以上品牌175个，国家高新技术企业数超过1000家，全社会研发投入占GDP比重2.6%。统筹推进协调发展，加快集聚区域竞争新优势。优化“东水西山一路连、南江北湖一河牵”的中心城市发展格局，基本建成广陵新城，完成生态科技新城、“三河六岸”区域基础设施建设，完善提升西区新城、南部新城。加快发展县城镇，特色发展重点中心镇，精心打造20个特色小镇，推进沿江沿河联动发展。到2021年城镇化率达70%。深入推进绿色发展，精心打造秀美扬州。倡导绿色低碳生活方式，建设国家循环经济示范城市。实施公园体系建设行动，全面建成10大生态中心。到2021年，PM2.5年均浓度五年下降20%，地表水省控以上断面优于Ⅲ类水质的比例达到75%。全市土地开发强度控制在21%以内。高水平推进开放发展，着力提升经济国际化水平。完成“510”外资并购行动计划、新一轮“530”招商行动计划。全面推进共享发展，提升市民的幸福感和满意度。

三是坚持把落实大战略、主攻大项目、构建大交通作为建设新扬州的强大支撑。主动全面融入“一带一路”、长江经济带、扬子江城市群、跨江融合发展和宁镇扬一体化等国家和省重大发展战略，加快形成区域融合发展新优势。突出效益为先，坚持“项目为王”，扎实推进供给侧结构性改革，持续推动每年新开工120个重大产业项目的任务落实，尤其是在100亿元、50亿元重特大产业项目方面实现新突破。加快构建现代综合交通体系，完善城市快速路网，推进328国道改造、京沪高速扩容及南延，建成连淮扬镇铁路扬州段及综合交通枢纽。加快扬马、宁扬城际等轨道交通过江通道规划研究和前期工作，推动北沿江高铁、京沪高铁宁扬联络线取得突破性进展。深化研究城市地铁规划、建设。实施扬州泰州国际机场提档升级工程。加快扬州港建设和港口资源整合。推进坚强智能电网建设。

四是坚持把系统化思维、项目化推进、精细化实施作为建设新扬州的有效保障。围绕市第七次党代会确定的“十件大事”，按照“五可”要求，一项一项抓推进、一件一件抓落实，全面提升政府工作的协调性、精准性和组织程度。在工作重点上，着力推进六大攻坚工程：聚力聚焦“12345”创新发展工程、美丽中国的扬州样板工程、健康中国的扬州样本工程、构筑多层次人才体系工程、县域经济提升工程、江淮生态大走廊建设工程。在工作路径上，制定实施六大行动计划：1.创新发展行动计划，推动全国“两创”基地城市示范和三大创新板块取得实质性进展，努力成为省“一中心一基地”重要增长极；2.“大学生城”建设行动计划，倾力打造创新人才高地，加快实现100万人才总量目标；3.县域经济振兴行动计划，不断缩小地区发展差距，着力推动县域综合实力争先进位；4.特色城市建设行动计划，持续深度挖掘大运河申遗价值，凸显文化生态两大优势，承办好第十九届省运会、第十届省园博会和2021年世界园博会，加快建设国际文化旅游名城；5.民生幸福行动计划，聚焦居民收入增长，提升基本公共服务惠民能力，建设全民共建共享的健康快乐家园；6.社会建设水平提升行动计划，健全基层社会治理体系，完善现代城市治理机制，巩固和谐社会建设水平。

2017年政府主要工作

今年是党的十九大召开的喜庆之年，是深入贯彻落实省、市党代会精神的起始之年，也是积极筹备省运会、省园博会的决胜之年。政府工作既面临保持经济稳定增长的发展任务，也面临推进供给侧结构性改革的转型压力；既面临多个发展战略规划的融合叠加，也面临区域经济地理重塑的竞争挑战；既面临彰显文化与生态特色的倒逼要求，也面临推进城乡居民持续增收的发展重任。

根据中央“稳中求进”工作总基调，也立足为转型升级留出较大空间，全市经济社会发展主要预期目标是：GDP增长8%，一般公共预算收入保持正增长，全社会固定资产投资增长12%，社会消费品零售总额增长10%，居民消费价格指数不高于省定目标，全社会研发投入占GDP比重2.4%，城乡居民人均可支配收入与经济发展同步增长，完成节能减排省定目标。

重点抓好以下八个方面工作：

1.振兴壮大实体经济，进一步推进产业集聚、拉长产业链条、提升产业能级，筑牢经济发展稳定向好的基本面

强化企业发展主体地位，大力弘扬企业家精神。落实“2号文件”要求，靠实举措，提振企业发展信心。持续监测实体经济运营质态，支持存量企业提升产品质量、稳住市场份额，守住经济基本面。引导培育经济增长新动能，实施“专精特新”培育计划，推动200家以上企业实现核心装备“数控一代”全覆盖。推进质量强市，制（修）订国际、国家、行业和地方标准20个以上，培育知识产权密集型企业50家，创省级以上品牌30个。加强企业家人才梯队建设，选树青年企业家和“创二代”先进典型，营造尊重企业家、有利于创新型企业家发展的社会氛围。

扩大有效投入，推进重大项目建设。提高投资效益，增强重大项目落地率、产出率。全年新开工重大工业、服务业、农业项目分别达50个、40个、30个，重点抓好21个列省重大项目，工业招商项目比重达60%以上，工业投资占固定资产投资比重提升至55%。大力招引民营企业500强项目，激发民间投资潜能。强化金融服务创新，持续推出金融产品，缓解企业融资难题。构建第三方指标为主的项目绩效评价体系，工业技改投入增幅17%以上，全市净增工业列统企业100家。

突出智能化发展方向，打造高端制造业新能级。实施智能制造“四个一”提升工程，逐步构建以智能制造为重点的新型制造体系，创成1个省级高端装备示范和特色产业基地，新建成智能车间10个以上，工业机器人保有量达2000台（套）。开展“特色高端装备引领行动”，大力发展以数控机床、粮食机械、节能环保机械、建材工程机械等四大领域为主要支撑的装备制造业，实施激光机床、精密液压、特种电缆等重大项目10项以上。着力推动汽车及零部件产业发展，壮大省级乘用车生产基地规模，推进省级新能源汽车产业基地建设。加快船舶产业转型升级，大力提高高技术船舶及海工装备开发生产能力。打造智慧化工园区，提升绿色化发展水平。提档升级新能源、半导体照明、智能电网、高端新材料研发与应用等优势产业，培育发展新一代信息技术、新医药等新兴产业，战略性新兴产业增加值占GDP比重16.5%。

放大集聚优势，加快发展现代服务业。优先发展生产性服务业，实施生产性服务业“十百千”工程，充分发挥生产性服务业在研发设计、流程优化、市场营销、物流配送、节能降耗等方面的引领带动作用。大力发展软件和互联网产业，促进业务收入年增长30%。科技广场建成运营，建立科技创新服务联盟和技术转移联盟，科技服务业收入突破80亿元。着力发展现代金融业，实现各类直接融资200亿元以上，加快扬州金融集聚区建设。推进发展航空物流，货邮吞吐量力超8000吨。出台并实施服务来扬旅游旅行者“3号文件”，提升全域旅游发展水平，推动东关街申创5A级景区、瘦西湖申创国家级旅游度假区，推进红色旅游、乡村旅游发展，旅游业增加值占GDP比重7.7%。积极发展文化创意产业，促进文化与金融、科技的深度融合。实施“互联网+商贸”行动计划，电商交易额增长30%。促进房地产市场平稳健康发展，保持市区房价稳定。服务业增加值占GDP比重46%以上。

着眼提质增效，推进现代农业建设。以农业供给侧结构性改革为抓手，推动农业多元复合经营，提升农业质量和效益。继续实施粮食绿色增产“1120”工程，新建粮食仓库10万吨以上。扎实推进农业生产全程机械化。建设6个50亿元产值连片特色产业基地。新建高标准农田15万亩以上。加快国家农业科技园区建设。开发推广新品种、新技术、新模式30项，新增“三品一标”农产品130个以上。深化农村产权制度改革，全面完成农村土地承包经营权确权登记颁证工作。加大农业招商力度，每个县（市、区）新招引1家农产品精深加工企业、新上1个投资1000万元以上农产品加工技改项目，新增市级以上农业龙头企业10家。省级以上农业龙头企业销售收入增幅8%。观光休闲农业年营业收入增长10%。在重视特色产业发展的基础上，新增农村电商销售企业60家。

2.持续推进创新驱动、改革牵引，全面增强经济发展内生动力，加快构建发展新优势

强化科技创新，充分激发经济增长新动能。启动新一轮创新型城市建设，推进全国“两创”基地城市示范建设三年行动计划。实施重大科技项目“双百”工程、“两化”融合提升工程、知识产权密集型产业培育工程，高新技术产业产值占规上工业比重45%以上。推进三大创新板块建设，新开工建设100万平方米科技产业综合体和众创空间，入驻企业总数超过1600家。加大创新企业培育力度，新增国家高新技术企业105家、省级以上“三站三中心”50家。持续推进开放创新，探索离岸孵化机制，引进知名高校和企业研创中心30家以上。扎实推进“绿扬金凤”“英才培育”等重大人才工程，引进100名高层次领军人才、1000名支柱产业发展急需专业技术人才和10000名基础性人才。

全面优化功能载体质态，提升园区集聚度、贡献率。做大做强市开发区半导体照明产业园等13个特色产业园。继续深化南北合作共建，各省级以上开发园区承接上海、苏南亿元项目3个以上。江都开发区争创国家级开发区，生态科技新城、高邮湖西新区打造具有竞争力的高新技术产业基地。健全市级开发园区考核评价淘汰竞争体系，推动省级以上开发区、高新区主要经济指标在全省位次前移，在全市经济总量中的比重提升。

高度重视招商引资，加速经济国际化进程。鼓励各地出台个性化、专业化、精准化的招商引资政策。全年实际利用外资12亿美元，新引进世界500强项目6个、重大外资并购项目2个，新签约央企合作工业项目15个。实施外贸快进快出战略，高新技术产品出口占比超过15%。鼓励企业“走出去”，全市外经营业额同比增长10%。争取10个较大规模的国际会议、赛事和活动来扬举办。扬州泰州国际机场旅客吞吐量增长15%以上。

深化供给侧结构性改革，推动重点领域改革取得突破。继续推进落实“三去一降一补”重点任务，有序关停、搬迁落后产能和低端劣质企业，削减钢铁产能80万吨。深入推进跨江融合发展综合改革试点。打造“互联网+公共服务”平台体系，加快建设线上线下、虚实一体的政务服务“一张网”。高邮市、扬州经济技术开发区开展相对集中行政许可权改革试点。市级公共卫生、农业、水利等领域推行“一个部门一支队伍管执法”试点，仪征市、广陵区整合组建综合执法队伍。整合城镇居民基本医疗保险和新型农村合作医疗（新农合）制度，建立“六统一”体系。完善国有资产管理体制，大力发展混合所有制经济。深化投融资体制改革，积极引导民间资本参与PPP项目。深化公立医院改革，全面建成运行县乡村一体化医联体。

3.聚焦以人为核心的新型城镇化，构建协同融合、紧凑有序的空间体系，在更高层次上统筹城乡区域发展

强化功能定位，持续优化城镇空间布局。围绕“一核多组团”，整体推进古城保护和利用，完善南河下等片区服务配套功能，江广融合区规划建设“七河八岛”和“三河六岸”滨水工程，西区新城改造提升明月湖周边区域，南部新城推进三湾公园、瓜洲旅游度假区等片区建设，蜀冈-瘦西湖景区加快实施唐子城保护和利用等项目。加快宝应、高邮、仪征老城区改造。继续实施高邮市和邵伯、月塘、氾水等镇国家新型城镇化综合改革试点项目。市县联动、分层分级，启动首批10个特色小镇建设。

立足资源禀赋，培大做强县域经济。壮大优势产业，推动县域差异化发展。引导邗江机床制造、宝应输变电装备产业向高端制造提升，促进江都船舶特钢、高邮电线电缆产业向“专精特新”方向发展，加快仪征国家级品牌轿车和零部件生产基地、广陵信息服务产业基地建设。宝应、高邮南北共建园区重点招引补链扩链项目，邗江环保产业园争创国家级特色产业基地，汽车产业园、安宜创业园加快创建省高新区，促进乡镇工业集中区特色化、集群化发展。放大文化生态优势，实施宝应园艺作物标准园和设施农业园项目，推动环高邮湖和邵伯、湾头、瓜洲等地旅游资源开发。仪征加快省园博会主展区、主展馆和园区内外道路管网、景观等基础设施建设，提升“展、住、行、游”等综合配套能力。

推动城乡一体，提升新农村建设水平。加快基本公共服务向农村延伸，统筹推进新农村建设。新改建农村道路380公里、危桥50座，镇村公交覆盖率提升至83%。疏浚河道50条，整治河塘300（条）面，新增高效节水灌溉面积4万亩。新创成省级“美丽乡村”10个、省级绿化示范村50个。培育农村集体资产股份示范合作社40个。扶持沿河、沿江地区65个市级经济薄弱村集体经济年收入分别达40万元、50万元。

4.坚持生态优先理念，加快绿色低碳循环发展，持续推进生态财富和物质财富同步累积

抢抓战略机遇，推进江淮生态大走廊建设。科学谋划江淮生态大走廊扬州段总体布局，重点实施沿线黑臭河道整治等27个项目，开展宝应湖、高邮湖、邵伯湖良好湖泊保护行动，实施“三退三还”工程，退渔还湖面积6.5万亩，建设生态防护林3000亩。全面完成集中式饮用水源地整治。县级以上城市污水处理厂出水全面达到一级A排放标准，工业集中区污水实现全收集全处理，确保南水北调水质稳定达到Ⅲ类。

坚持高点定位，加快生态中心和公园体系建设。突出生态中心建设中生态用地占比、生态廊道绿化率等指标，融入生态旅游、休闲度假等元素，提升经营管理水平。全面完成生态体育休闲公园体系“111”工程。注重植造珍贵、彩色树种，提升城乡绿化水平，全市新增植树500万株、成片造林2万亩，林木覆盖率23.4%，市区新增绿地100万平方米。

顺应群众需求，狠抓重点环境问题治理。坚决完成中央环保督察反馈问题年度整改任务，扎实开展“两减六治三提升”专项行动。淘汰化工落后产能，压降燃煤发电和非电用煤，PM2.5年均浓度比2013年下降20%，空气质量优良率70%以上。实施入湖河流水环境综合整治和农业面源污染控制工程，完成汤汪、高邮海潮等污水处理厂提标改造，推进“河长制”长效管护。完成150个行政村污水处理设施建设。启动污染场地土壤综合治理与修复。完成禁养区畜禽养殖场搬迁。规范沿江危化品码头运行管理。新建宝应垃圾焚烧厂。推行排污权有偿使用和交易。

5.主动融入国家和省重大发展战略，放大区位优势，提升功能品质，进一步增强城市持续发展能力

主动对接承接，推进宁镇扬一体化发展。以快联快通为目标，改扩建328国道，加快推动北沿江高铁、京沪高铁宁扬联络线等重大交通项目前期工作。以便民惠民为根本，完善宁镇扬三地医疗保险异地就医联网结算和社会保险关系顺畅转移接续机制。以功能互补为原则，强化与宁镇两地在旅游、科研、教育、卫生等领域的全方位合作。以产业融合为方向，加快宁扬毗邻地区融合发展，打造仪征-六合353省道沿线健康养老生态休闲带，积极承接江北新区汽车、电子、新材料等产业。

构建立体交通体系，全面加快重大基础设施建设。加快推进连淮扬镇铁路、东部交通枢纽、城市南部快速通道、金湾路等重点工程。开工建设五峰山过江通道公路接线、涉铁路段京沪高速扩容、扬子津路等骨干工程。完成江广高速改扩建路基工程，宿扬高速建成通车。新建G345仪征段、S331宝应段、S333高邮东段等国省干线，建成S611沿湖大道，S352江都段主体完工。实施扬州泰州国际机场一期扩建工程，建成国际货运区。完成淮河入江水道整治，继续推进长江镇扬河段三期工程建设，启动长江堤防提标加固和通扬线扬州段航道整治项目。

完善功能布局，增强城市承载力、辐射力。修编城市总体规划和土地利用规划，推进各类空间性规划“多规合一”。全面提升城市设计，强化文昌路、江阳路沿线规划控制。加快实施主要道路、交叉口、重要节点的园艺化改造和城市南部快速通道沿线改造提升工程。持续推进“不淹不涝、清水活水”城市建设。实施明月湖清淤和沿山河疏浚工程，建成瓜洲外排泵站主体基础工程。推行城市污水管网规划、建设、监管、养护“四统一”，坚持截污控污为先，新铺设污水管网42公里。开展省“公交优先示范市”建设，积极申创国家“公交都市”，新购新能源或清洁能源公交车200辆，新辟调整公交线路10条。推进安全（消防）通道、救护避灾通道和公共空间建设。试点推进综合管廊建设，加大地下空间开发力度。

推进市县联动，全面提升城市治理水平。立足常态化长效化管理，实施“五提升一整治”行动，确保通过文明城市国家复查测评。提升城区街面秩序。取缔市区马路市场17处，推动出台《扬州市违法建设防控与查处条例》。提升环卫保洁质效。主城区街巷保洁市场化作业实现全覆盖，道路机扫率90%，各县（市）城区道路机扫率提高5个百分点。提升街景容貌。综合整治市区80条路段，美化出新14条路段“城市家具”，清理旅游景点、高速道口、县城区中心地带“六乱”现象。提升数字化城管效能。将江广融合地带纳入主城区数字化城管监管范围，推动县（市）数字化城管系统规范运行。提升执法管理能力。推进执法事项属地化管理，向乡镇（街道）下移执法重心，主城区实现派驻执法全覆盖。整治道路交通秩序。突出学校、医院、农贸市场及大型商业综合体周边等重点区域，狠抓八类交通违法行为整治。

6.普惠发展社会事业，优化区域资源配置，不断推进基本公共服务均等化

举全市之力，扎实做好省运会各项筹备工作。加快省运会体育场馆建设，建成开放游泳健身中心、射击运动中心，完成市体育公园改造提升工程，推进明月湖和体育公园的连通改造。开展比赛场馆周边环境综合整治，实施重要道路沿线及可视范围内的立面改造。加强备战训练，确保本届省运会综合排名处于历史最好水平。积极办好鉴真国际马拉松赛、环高邮湖国际自行车越野赛等其他赛事。

加快发展医疗卫生事业，全方位、全周期保障群众健康。开展十大主题健康促进行动。启动创建全国基层中医药工作先进单位。推进仪征、宝应人民医院创建三级医院，高邮人民医院争创三级乙等医院，异地新建江都人民医院。全部建成18家农村区域性医疗卫生中心。以村（社区）为单位家庭医生签约服务实现全覆盖。加强食品药品安全监管，中小学食堂推进“透明厨房”建设。实现乳制品、肉类、食品添加剂等生产企业电子追溯系统全覆盖。健全计生家庭奖励扶助机制。

立足学有优教，公平优质发展各类教育事业。推进教育现代化建设，新创省义务教育现代化学校50所。新改建幼儿园10所，建成8所中小学。高考普通类本科上线率保持全省前列。办好职业教育集团，启动扬州技师学院迁建。改善特殊教育办学条件。加强与扬州大学等驻扬高校深度合作。探索建设大学生实习实训基地。

统筹发展文化事业和文化产业，增强城市软实力。完善公共文化服务体系，建成331个村（社区）综合性文化中心，新开设城市书房10家以上。加大非遗人才培养力度，创作扬剧、扬州弹词等一批优秀文艺作品。施行《大运河扬州段世界遗产保护办法》，实施淮扬运河主线槐泗段等大运河遗产保护项目，推进隋炀帝墓、龙虬庄考古遗址公园建设。大力发展文化产业，实施凤凰水街、宝应泰山殿历史文化景区等文化旅游项目，加快建设上海（扬州）网络视听产业园，推进486等非遗产业园区融合发展，文化产业增加值占GDP比重5%以上。

7.注重共建共享，多措并举加快富民步伐，让群众拥有更多获得感

注重创业带动就业，大力拓宽居民增收渠道。实施促进充分就业行动。城乡劳动者职业技能培训3万人次，城镇新增就业5万人，转移农村劳动力1.1万人，期末城镇登记失业率控制在4%以内。强化创业信息资源和平台整合，加大财政资金支持和统筹力度，完善专业化、网络化创业服务体系。新建创业指导中心9个、市级创业孵化基地10个，发展10个专业化众创空间，开展创业培训1.5万人，支持成功创业7500人，新增就业创业20万人次以上。降低小额担保贷款门槛，丰富创业融资模式。鼓励农民工返乡创业，新增生产经营型、专业技能型和专业服务型农民1.5万人。加快建筑业做大做强，推进农民增收。

稳步提高社会保障水平，筑牢民生底线。落实各类社保政策，发挥全民参保登记成效，市区新增养老保险5000人。继续推进机关事业单位养老保险制度改革。引导社会资本进入养老服务业，启动编制市区养老设施布局规划。全民医保覆盖率98%以上。推进被征地农民刚性进保。严格执行残疾人两项补贴制度。落实孤儿保障标准增长机制。推进自然灾害保险全覆盖。

努力办好12项重点民生实事，全面提升居民幸福指数。（1）交通治堵方面，积极打通“断头路”，完成念泗路西延工程，启动江平路西延、真州路北延、大学路南延等建设；促进交通微循环，拓宽改造瘦西湖路与江平东路交叉口、扬子江北路与扬州北高速出入交叉口、新328国道与渡江南路交叉口等交通堵点。（2）有序停车方面，建设智能停车系统，实施市区停车收费管理一卡通。市区新建公共停车场2座，在城市东、南、西、北出入口分别设置大型临时换乘中心。每个县（市）新建公共停车场2座以上。（3）食品安全方面，市区新改扩建农贸市场10个，重点改建、新建以叶菜类为主的“菜篮子”工程基地2000亩以上，创建标准化养殖示范场10个。（4）环境保护方面，完成七里河等22条黑臭河道整治，宝应、高邮、仪征、江都至少完成1条城区主干河道整治；关闭搬迁844个规模化畜禽养殖场和106个小船厂、砂石场；关停36家化工企业，加快推进扬农、通裕、联环药业退城进园。（5）“八老”改造方面，全市整治老小区52万平方米，市区完成“城中村”改造15个，具备条件的老小区和老城区通上天然气。（6）住房保障方面，市区实施公共租赁住房（含廉租住房）保障450户、经济适用住房保障300户；全市棚户区改造新开工12300套，基本建成11000套。（7）精准扶贫方面，以人均年收入7000元以下低收入农户全部脱贫为目标，宝应、高邮、仪征城乡低保标准以县为单位实现同标，全部达到580元/月以上。（8）文化惠民方面，建成广陵公共文化中心、扬州戏曲园，推进新大剧院建设。（9）教育提升方面，完成中小学校安工程8.6万平方米，建成广陵学院新校区，江苏旅游职业学院建校招生。（10）医疗卫生方面，启动建设市妇女儿童医院、公共卫生中心，改扩建市传染病医院。（11）社区建设方面，市区探索打造高标准邻里中心3个，建设颐养示范社区9个。（12）公共安全方面，市区改扩建8个公安派出所业务用房，在视频盲点、治安复杂地区新增1000个高清探头。建成广陵新城消防站。

8.彰显公平正义，切实加强和创新社会治理，进一步提升社会文明程度

注重软硬件建设，夯实基层基础工作。巩固村（居）减负增效成果，推进“三社”融合发展，探索社区服务社会化。加大政府购买社会服务力度，积极培育社会组织，提高承接公共服务和社会公共事务的能力。整合各类信息系统，打破“数据烟囱”“信息孤岛”，打造大数据服务平台，启动建设“云上扬州”。落实信用联动奖惩政策，推动“诚信扬州”建设。

强化综合治理，维护社会和谐稳定。推动人民调解、司法调解、行政调解和协会民间调解联动。完善立体防控网络，建设“反电信网络诈骗犯罪中心”，依法打击涉众非法集资和互联网金融领域的各类犯罪。健全应急预案体系，强化灾害监测预警和风险防控。做好社区矫正与安置帮教。严格落实安全生产责任，防止重特大安全事故发生。支持国防和军队改革，深入实施军民融合发展战略，推进“八创”全国双拥模范城。

坚持法治引领，营造良好法治环境。建立法治政府建设报告制度。加强地方立法，健全立法项目公众征集机制。科学实施“七五”普法规划，落实“谁主管、谁普法”责任制。进一步强化依法行政，继续完善行政执法与刑事司法衔接机制。构建覆盖城乡的公共法律服务体系。

全力建设人民满意的政府

各位代表，本次人代会将选举产生新一届政府。新一届政府将牢固树立“四个意识”，特别是核心意识、看齐意识，统筹推进“五位一体”总体布局和“四个全面”战略布局，贯彻落实新发展理念，践行“两聚一高”，办好“十件大事”，努力建设“强富美高”新扬州。为此，我们必须时刻牢记职责使命，时刻坚守为民情怀，时刻秉持严实作风，全力建设规矩规范、开明进取、廉洁高效、人民满意的政府。

强化权责法定，打造法治政府。严格执行《法治政府建设实施纲要》，把政府工作全面纳入法治轨道，依法界定全面、清晰的政府权力清单，推动政府及其组成部门职责法定化。自觉接受市人大法律监督、工作监督和市政协民主监督，健全审计监督、社会监督、舆论监督机制。加强对议案建议和提案办理考核，切实提高办理质量。落实重大行政决策合法性审查制度。发挥好政府法律顾问作用，完善第三方评估工作机制。规范行政执法行为，推动县（市、区）全部设立行政复议委员会。以政府诚信建设推动商务诚信，带动全社会诚信体系建设。

强化务实高效，打造服务型政府。构建政府、企业、群众“命运共同体”。更大力度培植实体经济，提高服务新兴经济市场主体的快速响应能力。进一步简政放权，深化“放管服”改革，加大“中梗阻”治理，持续推动权力下放、服务下沉，切实为服务基层“清障搭台”。办好政府服务热线和《寄语市长》，健全新闻发布制度。坚持民之所望，施政所向，把群众满意作为第一标准，着力打通政策落实、为民服务“最先和最后一公里”，提高基层和群众对政府工作的满意度。

强化开明开放，打造创新政府。抓创新就是抓发展，谋创新就是谋未来。新一届政府必须始终保持改革开放的锐气和勇气，加快构建更具活力的体制机制。进一步调适责权关系、事权关系，充分调动市与区（县）、市与部门多个积极性。进一步改进作风，不因循守旧，不固步自封，以进取开新篇，以实绩论英雄。更多运用市场机制和手段推动发展，更好发挥市场在资源配置中的决定性作用，推动“大众创业、万众创新”蔚然成风、生机勃发。

强化职责使命，打造担当政府。应对经济转型新挑战，赢得区域竞争新优势，政府必须主动作为，机遇面前不迟疑，矛盾面前不回避，困难面前不退缩，实干苦干，顶真较真，善作善成。健全正向激励和容错机制，加大对工作成效明显的地区和部门激励支持力度，让敢于负责、敢于担当、敢于碰硬的实干者有机会、有舞台；完善严格的考核机制，层层传递压力，对各类失职失责行为保持“零容忍”，严肃督查问责，让有令难行、遇事推诿、慵懒散漫的不作为者丢位子、受惩戒。

强化清正严明，打造廉洁政府。严格落实全面从严治党要求，认真履行“一岗双责”，坚持把纪律建设贯穿于政府工作各个方面。严格执行中央八项规定，驰而不息狠刹“四风”。厉行勤俭节约，严控“三公经费”，延伸“三直接”十大关键环节操作规范，强化工程招投标、质量安全、资金拨付等关键环节的风险防控。加强执法、效能、廉政监察，加大财政收支管理、专项资金管理和国有资产管理，严格控减政府债务，完善预算管理机制和程序，提高财政资金使用绩效，把有限的资金和资源更多地用在社会发展和民生改善上。

各位代表！时代赋予重任，人民寄予厚望。让我们更加紧密地团结在以习近平同志为核心的党中央周围，在中共扬州市委的正确领导下，凝心聚力、开拓进取、攻坚克难、奋发有为，以优异成绩迎接党的十九大胜利召开，为“迈上新台阶、建设新扬州”、谱写好中国梦第一个百年梦想的扬州篇章而努力奋斗！
